# AWANet: Attentive-Aware Wide-Kernels Asymmetrical Network with Blended Contour Information for Salient Object Detection

**DOI:** 10.3390/s22249667

**Published:** 2022-12-09

**Authors:** Inam Ullah, Muwei Jian, Kashif Shaheed, Sumaira Hussain, Yuling Ma, Lixian Xu, Khan Muhammad

**Affiliations:** 1 School of Computer Science and Technology, Shandong Jianzhu University, Jinan 250101, China; 2 School of Computer Science and Technology, Shandong University of Finance and Economics, Jinan 250014, China; 3 School of Computer Science and Engineering, South China University of Technology, Guangzhou 510006, China; 4 Visual Analytics for Knowledge Laboratory (VIS2KNOW Lab), Department of Applied Artificial Intelligence, School of Convergence, College of Computing and Informatics, Sungkyunkwan University, Seoul 03063, Republic of Korea

**Keywords:** salient object detection, saliency detection, attention recognition, attention mechanism, multi-scale, asymmetric convolutions

## Abstract

Although deep learning-based techniques for salient object detection have considerably improved over recent years, estimated saliency maps still exhibit imprecise predictions owing to the internal complexity and indefinite boundaries of salient objects of varying sizes. Existing methods emphasize the design of an exemplary structure to integrate multi-level features by employing multi-scale features and attention modules to filter salient regions from cluttered scenarios. We propose a saliency detection network based on three novel contributions. First, we use a dense feature extraction unit (DFEU) by introducing large kernels of asymmetric and grouped-wise convolutions with channel reshuffling. The DFEU extracts semantically enriched features with large receptive fields and reduces the gridding problem and parameter sizes for subsequent operations. Second, we suggest a cross-feature integration unit (CFIU) that extracts semantically enriched features from their high resolutions using dense short connections and sub-samples the integrated information into different attentional branches based on the inputs received for each stage of the backbone. The embedded independent attentional branches can observe the importance of the sub-regions for a salient object. With the constraint-wise growth of the sub-attentional branches at various stages, the CFIU can efficiently avoid global and local feature dilution effects by extracting semantically enriched features via dense short-connections from high and low levels. Finally, a contour-aware saliency refinement unit (CSRU) was devised by blending the contour and contextual features in a progressive dense connected fashion to assist the model toward obtaining more accurate saliency maps with precise boundaries in complex and perplexing scenarios. Our proposed model was analyzed with ResNet-50 and VGG-16 and outperforms most contemporary techniques with fewer parameters.

## 1. Introduction

Salient object detection (SOD) aims to distinguish the most discernible and eye-catching locations in an image. Most humans can quickly detect what draws their attention in an image. However, this is a strenuous task in computer vision. The saliency detection problem involves two sub-categories: eye fixation and salient object detection. We focused on SOD. Unlike segmentation methods, which classify all foreground object pixels with a classification label, SOD tasks selectively process the most eye-catching region/object in a scene and drastically reduce the computational cost.

It has typically benefited several computer vision activities as a preprocessing step. Examples include object recognition [1,2,3], content-aware image editing [4], resizing [5], visual tracking [6,7], person re-identification [8,9,10], image retrieval [11], and video summarization [12].

Motivated by cognitive research on visual attention [13,14], previous studies have mostly focused on the evidence that contrast [1] contributes to saliency detection. These approaches mostly consider handcrafted local and global contextual features and their mutually learned weights. Local contextual features help locate object boundaries, and global contextual features help capture the abstract details of the salient object in terms of texture, color, and intensity. Although these previous heuristic-based methods have demonstrated their importance, they lack high-level semantic knowledge for extracting salient regions in a simple image background, which frequently restrains the power of feature extraction when handling images with a complex background. SOD must extract all required features instead of relying only on hand-crafted low-level features.

**Figure 1:**
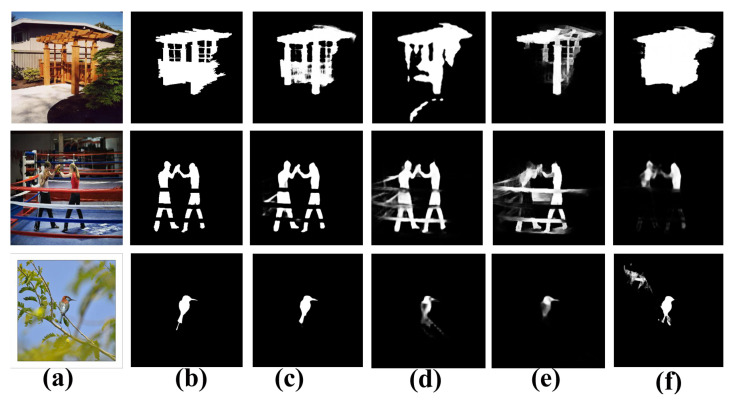
Sample comparisons of our method with others. From left to right: (**a**) input image, (**b**) ground truth, (**c**) saliency map of our approach, (**d**) saliency map of CPD [21], (**e**) saliency map of AFNet [22], and (**f**) saliency map of R3Net [23]. Our method generates more rigorous saliency maps with defined boundaries.

Recently, convolutional neural networks (CNNs) [15] have overcome the limitations of conventional handcrafted features. In particular, after the development of fully convolutional neural networks (FCNs), promising results have been achieved. However, they continue to encounter two significant challenges in SOD. (1) Contexts play a vital role in SOD [16,17,18,19,20], i.e., high-level contexts depict relationships between salient regions/objects and are thus useful for learning the overall positions of salient objects, whereas low-level contexts ensure fine detail features used to depict the boundaries of salient objects. However, learning the contextual representation for objects of varying scales and sizes within complex scenarios poses a significant challenge for SOD models. (2) Obtaining estimated results with acute boundaries is difficult because of repeated upsampling and pooling operations. Figure 1 shows that different methods encounter problems while localizing the complex salient regions and borders for objects of varying scales.

To handle these problems, we propose an attentive-aware wide-kernels asymmetrical network with blended contour information (CI) for SOD (AWANet) that can produce high-quality saliency maps. AWANet contains three efficient and effective modules: a dense feature extraction unit (DFEU), cross-feature integration unit (CFIU), and contour-aware saliency refinement unit (CSRU). To address the first problem, additional contextual information must be explored. A common technique is to employ convolutions with large kernels or stack multiple local convolution layers to perceive contexts in a large field of view [20,24]. Atrous convolutions [24] with high dilation rates are often used to increase the receptive field. Nevertheless, large kernel dilation filters tend to result in a high computational complexity and consume considerable GPU memory. Sparse sampling in atrous convolutions with a large dilation rate produces a gridding problem [19] and may be unable to sufficiently capture local or contextual information. To overcome these constraints, we designed the DFEU by introducing asymmetric large kernel convolutions into the final four stages of the backbone. We ignore the first level for subsequent operations to avoid the burden of a large parameter size owing to its large resolution size. The proposed DFEU follows a novel mechanism by introducing asymmetric large kernel sizes and grouped-wise convolutions with channel reshuffling that can extract more semantically enriched features for each resolution with comparatively fewer parameters. The DFEUs first divide the available channels of the specific resolution into two sub-resolutions before performing group convolution and global context block (GCB) operations. The GCB block introduces parallel asymmetric convolutions with different large sizes: (k×1)(1×k) and (1×k)(k×1) kernels for each resolution resulting in large fields-of-view with fewer parameters. The DFEU integrates the downsampled features for subsequent processing and employs channel reshuffling with channel reduction. After the DFEUs, a careful fusion method can integrate each resolution’s complementary features to obtain accurate saliency maps. However, the FCN-based levels always contain significant discrepancies, and the feature blending often degrades the model performance. Moreover, the subsampling operations on one side can explore more semantic features but cause the loss of influential low-level details, which cannot be recovered in subsequent operations.

To address the aforementioned problems, we designed CFIUs for each subsequent resolution after the DFEUs. The proposed CFIU performs three functions simultaneously, unlike contemporary feature integration modules. First, it simultaneously extracts different resolutions and performs sub-sampling to explore more semantic features. The sub-sampled branches depend on the specific position of the backbone level, and each branch employs channel attention with large asymmetric kernels to focus on the importance of different regions. The residual connection re-weighs the lost information after fusing the sub-sampled information, and channel re-shuffling further improves the performance without additional parameters. Second, it extracts high-level features and progressively increases the number of sub-sampling branches in a top-down movement for the lower level using dense short connections. This unique mechanism reduces the parameter size caused by the constraint expansion and prevents the model from the global feature dilution effect during top-down propagation. Third, it retrieves CI that can partially compensate for the low-level details lost during maximum pooling at the backbone. The proposed CFIUs can guide the model towards accurate predictions. However, for some complicated scenarios, the model has been observed to confuse between salient and non-salient regions owing to confused boundaries.

To address the ambiguity between complex background scenarios, some approaches have used edge labels to augment the training process of segmentation networks by including auxiliary boundary loss functions or designing unidirectional frameworks [25,26,27,28]. They utilize the edge features solely to enhance the representational capability of edge information. Previous research has established that combining edge features results in more precise segmentation maps. However, because edge features have not been well exploited in current edge-aware networks, imprecise edge features remain a challenge. Considering the worth of contour-based supervision, we propose using a CSRU on each output side to reduce the transition of irrelevant background noise and retain the object boundary. Unlike existing edge refinement modules, the proposed CSRU is progressively expanded in a similar manner as the CFIUs by retrieving the contour details directly from lower layers. Subsequently, by adopting deep supervision for each decoder stage, the learned information is fed to the next stage at the decoder side to determine the final, accurate saliency maps with more precise information. The large asymmetric kernels, with a contour-based spatial attention and constraint expansion mechanism, differentiate the designed CSRU’s effectiveness from existing methods. Our suggested method’s primary contributions are summarized as follows:

- We offer a unique light-weight DFEU capable of efficiently and effectively capturing rich contextual information to enhance the inter-resolutions of the backbone for more semantically enriched features.
- We designed a CFIU that sub-samples the specific resolution into different sub-resolutions according to the input features. The dense short connections of high and contour-based features with wide kernels, asymmetric convolutions, and channel-wise attention direct the model towards more rigorous and accurate saliency maps. In addition, a subsequent CSRU module was designed to improve the saliency map using dense contour-based short connections to strengthen and refine the saliency maps with precise boundaries for more perplexing and challenging scenarios.
- Our model is relatively smaller and more efficient than other large-scale backbone networks, such as ResNet-50 in the given research domain, with only 26.7 million parameters and a real-time speed of 31 frames per second (FPS). Experiments demonstrate our proposed approach’s superiority by analyzing the results on five challenging datasets and comparing 15 state-of-the-art methods.

## 2. Related Work

### 2.1. Salient Object Detection

In the early development stage, SOD methods typically used hand-crafted features, such as color contrast [29,30,31], background boundary [32,33,34], and center-surround priors [35,36]. Although these low-level models exhibited positive effects, their execution was imperfect for images with complex salient objects or clutter and complicated backgrounds. These models have a low computational efficiency and often destroy the underlying feature structure. See [37,38] for more details on previous and CNN-based methods.

Recently, owing to the outstanding achievements of CNNs in computer vision, deep learning has been established as an encouraging substitute for SOD tasks. Some initial deep learning models for SOD tasks use the CNN architecture to predict each image segment’s saliency score using object proposal [39] or superpixel [40] schemes. Wang et al. [39] suggested two neural networks for SOD tasks. One network was learned using a local patch to regulate pixel-wise saliency values. The other used the global feature to predict the saliency score for each object region. Liu et al. [18] proceeded with a hierarchical top-down pathway and embedded local and global modules to obtain all constructive information from the pixels. Li and Yu [40] first created several input image segments, and the neural network was trained for each segment separately. These networks were then combined and used several convolution layers. Zhao et al. [41] developed a multi-context deep structure with two branches that extracted local and global contexts and then integrated them.

### 2.2. Multi-Level Information

Several studies that used FCN-based methods [42] proved that the feature integration of multiple layers is advantageous for producing better results. Deep layers of the FCN network contain semantically contextual knowledge for recognizing the salient object’s location and category. In comparison, shallow layers encode fine spatial details for reconstructing the corresponding boundary of the salient object. Therefore, several works [43,44,45] have adopted multi-level features for SOD. Hou et al. [44] designed a model to incorporate multi-layer features utilizing a short connection in a top-down manner. Zhang et al. [43] integrated different level features at multiple resolutions to estimate saliency maps. Luo et al. [45] proposed a top-down refinement framework in which refined features propagate from deeper layers to shallower layers.

Similarly, Zhang et al. [46] utilized a bi-directional message-passing scheme by applying a gate procedure to monitor and regulate feature propagation among different layers. However, during multi-layer feature integration, some features interfere with each other. Combining the features of different layers to suppress the noise and boost the salient features, by leveraging a selective process, remains an important problem in saliency detection. Unlike the aforementioned methods, the proposed scheme integrates all deeper-level features to update the current resolution based on stronger contextual information.

### 2.3. Multi-Scale Information

The FCN [47] is considered the pioneering network that directly integrates features, from low- and high-level stages, to enhance the semantic segmentation accuracy. Similarly, the feature pyramid network (FPN) [48] and U-Net [49] pursued a top-down pathway to extract multi-scale features, from high- to low-levels, and sequentially integrated them. Deeplabv2 [24] employed an atrous spatial pyramid pooling (ASPP) module to extract multi-scale features with different dilated convolutions. Dense ASPP [50] enhances the ASPP with dense connections. Zhao et al. [20] adopted a pyramid pooling module (PPM) to integrate multi-scale contextual information with pooling operations. The PPM and ASPP are the two most common modules used for multi-scale feature extraction and are often applied at the deeper layers of the network [23,51]. Generally, the in-depth features of the FCN-based networks, at the topmost layers, cannot handle large-scale variations owing to lacking information. Hence, the PPM obtains multiscale features through multiple downsamplings. The ASPP obtains multi-scale features by enlarging the receptive field with different kernel sizes to successfully handle objects of various scales. However, they both lose the object’s spatial details owing to multiple downsamplings and minimize the connectivity among features by inserting additional holes [52,53]. Here, we propose a more robust method than that of contemporary multi-scale approaches to extract the multi-scale feature for each backbone level by introducing (k×1) and (1×k) parallel convolutions with a large kernel size, which can provide more contextual knowledge with fewer parameters.

### 2.4. Attention Mechanism

The attention-based models in recent neural networks that mimic the human visual system process have significantly improved on computer vision tasks. The main idea of the attention mechanism in neural networks is to allow the network to concentrate on the maximum significant parts and then weaken or enhance a large amount of the information selected. For instance, Hu et al. [54] applied a squeeze and excitation mechanism (SENet). The squeezing process compresses the feature by applying global average pooling, and the excitation mechanism obtains the weighted feature maps by applying two fully connected layers. This process significantly increases the precision of image classification models.

Moreover, Woo et al. [55] proposed the convolutional block attention Module (CBAM) model, which expanded SENet from a one-dimensional channel to a two-dimensional channel and combined the weighted feature maps of both the average and max-average feature maps. Liu et al. [18] designed a convolution and bidirectional long short-term memory (LSTM) and used a local and global pixel-wise attention mechanism, expanding the receptive field to mitigate errors. Few methods, such as [18,42,56], have used the attention mechanisms for SOD. However, our approach differs in that previous methods have typically utilized a single-attention design. Our approach follows a constrained module expansion according to the inputs received. The subsampled attentional branches independently observe the saliency importance of each object sub-region and then integrate them according to the guidance of contextual and contour-based information using residual connections.

### 2.5. Contour-Aware Modules

Recently, some studies have attempted to exploit additional boundary information by adopting contour labels for saliency detection to produce clear boundary saliency maps. In [45], Luo et al. used an additional intersection over union (IOU)-based edge loss to further define the boundaries of the predicted saliency maps directly. In [57], the authors combined multi-level convolutional features following recurrent and edge-based information guidance. Guan et al. [52] exploited the fine-tuning of the holistically nested edge detection (HED) [58] network for edge detection and integrated the complementary information with the saliency detection stream to predict the boundaries of salient objects. Zhao et al. [25] trained salient edge features by exploiting the IOU loss for salient object detection tasks. Wang et al. [26] also exploited edge-based features to refine the boundaries of saliency maps. Most existing methods have used boundary information to enhance accuracy, and few studies have focused on refining edge features. Unlike existing methods, we use a unique light-weight module to simultaneously extract high-level and contour-based information using dense short connections. The integrated information is split into two branches with channel- and spatial-wise attentions with large asymmetrical kernels to guide the model towards more precise and accurate saliency maps.

## 3. Proposed Method

**Figure 2:**
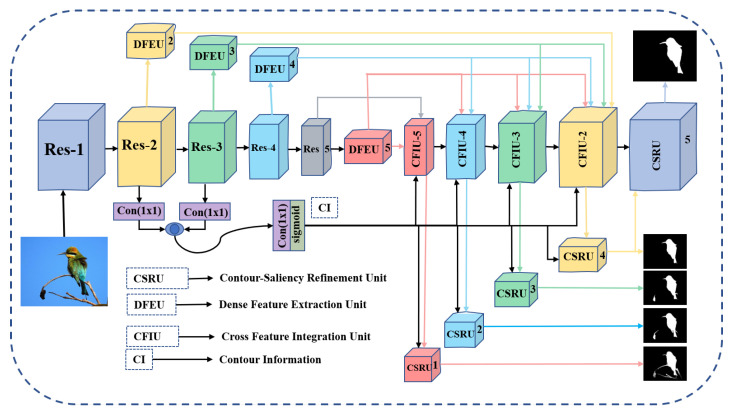
Overview of the proposed AWANet: attentive-aware wide-kernels asymmetrical network with blended contour information for salient object detection.

This section provides an overview of AWANet, which comprises three types of sub-modules: DFEUs, CFIUs, and CSRUs. Figure 2 shows the proposed method’s structure.

### 3.1. Overview of Network Architecture

Our model is based on the FCN encoder–decoder architecture with a pretrained VGG-16 or ResNet-50 backbone network. We first remove the last global average-pooling layer and fully connected layers of the backbones to achieve the saliency detection tasks. The input image dimension is reduced when propagated from the shallower layers to the deeper layers in the backbone. Therefore, feature maps at the last level of the backbone are subsampled 32 times as the input image. The feature maps of each backbone level, that is, n=2,3,…5, contain a spatial size of $H/2^{n}\times W/2^{n}$. The backbone extracts basic multi-level features and abstractions. The SOD images have significant variations in scale and locations; thus, the simple backbone network cannot handle these complexities with a cluttered background. Therefore, the DFEU is applied at different stages to boost the intra-layer capabilities and overcome gridding concerns caused by sparse connections by adopting wide and dense receptive fields. Then, at each step, the unit CFIU collects contextual features following the DFEU and contour-based information via short connections to avoid the dilution effect of low- and high-level features during bottom-up and top-down propagation. The sub-sampled constrained-wise attentional modules with wide range asymmetrical kernels enforces the model to note the object’s sub-regions importance. To further refine the saliency maps for perplexing and challenging scenarios, the CSRU is adopted in the same manner as the CFIUs. The CSRU extracts the high- and low-level contour-based information and then splits the integrated features by adopting channel and spatial-wise attention to generate more appropriate saliency maps with rigorous boundaries for complicated scenarios.

### 3.2. Dense Feature Extraction Unit (DFEU)

**Figure 3:**
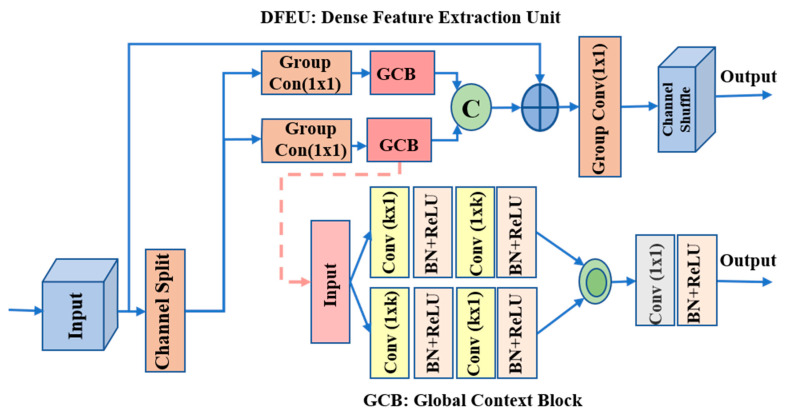
Dense feature extraction unit (DFEU) with large (k×1) (1×k) and (1×k) (k×1) kernel-size convolutions for k=3,…,11.

The SOD datasets were observed to contain different images of varied scales and locations. Owing to scale variability, a single-scale convolution has difficult detecting the correct size of salient objects. Various studies by Chen et al. [24] and Zhao et al. [20] used multi-scale feature extraction modules, such as the ASPP module and PPM, to obtain robust segmentation results. In addition, pyramid pooling module (PPMS) [20] uses parallel convolutions to observe more contextual knowledge, which loses local information and requires more parameters. In contrast, the ASPP module [24] contains different atrous parallel convolutional layers with varying dilation rates. The atrous convolutions enlarge the receptive fields. However, their sparse sampling and high dilation rates may not capture sufficient context information in the atrous convolution to provide an excellent performance for the specified task. We propose constructing a dense context extraction, i.e., the DFEU, to circumvent the sparsity and establish dense connections within a (k×k) receptive field. As shown in Figure 3, we are inspired by grouped convolution [59] and the channel shuffling in [60], which may provide significantly richer feature representational capabilities at a much lower cost.

Technically, each DFEU begins with splitting the input features into two lower dimensional branches. Each has half-input channels and applies a (1×1) grouped convolution. Then, each unit is followed by a GCB that utilizes two parallel branches, i.e., (1×k),(k×1) and (k×1),(1×k), by adopting spatially separable convolutions to efficiently capture the local region features. Following each convolution process, batch normalization (BN) with rectified linear unit (ReLU) functions are applied. Our GCB block provides a large receptive field without sparsity in the receptive fields to obtain broader context information while maintaining reasonable computational limits. Compared with ASPP and other dilated convolutions, it addresses gridding concerns.

The embedded GCB contains the different values of *k* for varying stages of the backbone. For example, for n=2,3…5, the value of k is fixed at (3,5), (5,7), (7,9) and (9,11). The two parallel (k×1) and (1×k) convolutions are merged by simple concatenation, and the resulting features are applied to a (1×1) grouped-wise convolution propelled by BN and ReLU functions. Similarly, the splitting branches of the DFEU are merged again by performing channel-wise concatenation and using a (1×1) grouped convolution to reduce the number of channels to 64 after resizing their input dimension. Because each unit concentrates on a particular aspect of a feature, information exchange is limited across all channels, which may adversely affect the object structure of salient regions. To resolve this, the DFEUs shuffle channels over the combined features to facilitate cross-channel information flow.

The proposed DFEU module is efficient and can help locate accurate salient regions. First, it enlarges the receptive field with a (k×k) region to extract more discriminative features for subsequent modules. Second, each DFEU randomly shuffles the aggregated feature channels to enhance the network capability without increasing the computational complexity.

### 3.3. Cross Feature Integration Unit (CFIU)

**Figure 4:**
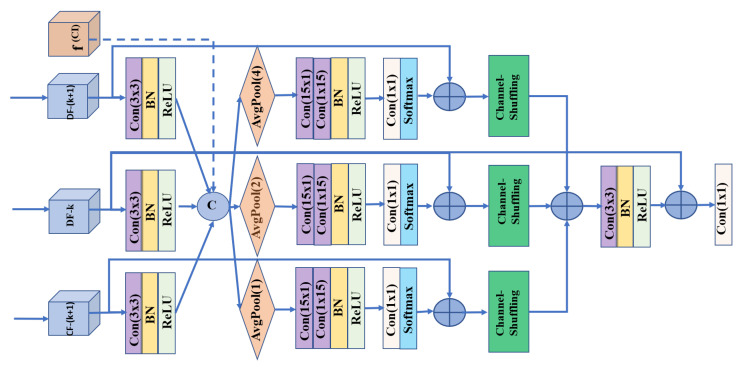
CFlU-k block structure. This example shows CFlU-4, which takes inputs from CF-(k+1), DF-k, and DF-(k+1). CF-k and DF-K denote CFIU and DFIU modules, respectively.

The backbone network contains different abstraction stages, in which each set includes a piece of specific semantic information for salient object recognition. High-level features include semantic knowledge because of the expanded field of view; hence, these features help recognize the contextual region of the image. Low-level features contain local and spatial information because of their small field of view. Therefore, the local information helps identify salient boundary regions. Based on this, we designed the CFIU to better utilize different resolution capabilities. The CFIU is a simple and effective module that can be integrated into any deep network to enhance feature representations. It maintains the multi-level strategy to integrate different feature representations after the DFEU and applies a stack of soft attention layers [26,61] with varying rates of downsampling [20] and learns to update them with residual connections and larger receptive fields. The proposed CFIU differs from existing modules in that it eliminates the dilution of contextual and low-level details during top-down and bottom-up propagation. The expansion of the sub-sampling branches is constrained according to the input received, providing semantically enriched features with a relatively lower computational cost. Let our encoder side contain k = 2 to N = 5 feature maps for further processing at their corresponding decoder side. Then, at the k^*th*^ stage, CFIU-k receives the output of its high-level CFIU-k+1 or top-level of the encoder as input features. Simultaneously, it obtains the high-level features from DFEU-k to DFEU-N. The DFEUs contain more enriched contextual features, thereby avoiding the feature dilution effect in top-down propagation with relatively more accurate object sizes. Figure 4 visualizes CFIU-4. It receives its inputs from the preceding CFIU-5, DFEU-4, and DFEU-5. Similarly, CFIU-3 receives inputs from CFIU-4, DFEU-3, DFEU-4, and DFEU-5. Moreover, as known, the low-level features weaken gradually in the bottom-up paradigm owing to upsampling and intrinsic convolution layers. The low-level features are essential for boundary information. The CI learns the boundary information by imposing joint edge-based supervision to retain the boundary information intact and can be expressed mathematically as follows:

(1) $$\begin{array}{cc} \; & CI=Sig\cdot Conv_{1}\left(Conv_{1}\left(L_{2}\right)⊙Conv_{1}\left(L_{3}\right)\right), \end{array}$$

where Sig, $Conv_{1}$, and $L_{k}$ represent the sigmoid function, (1×1) convolution and kth stage of the encoder, respectively. The CI is input into each CFIU block to guide them towards accurate boundary regions. Let our $CFIU_{k}$ block receive $CFIU_{k+1}$, $DFEU_{k}$, $DFEU_{k+1}$, ⋯, $DFEU_{N}$ feature maps. Each input is upsampled according to the given $k^{th}$ stage and then applied to a (3×3) convolution layer with BN and a ReLU activation function. Then, these features, along with contour-based features (CI), are integrated via channel-wise concatenation to create complementary feature maps $p_{k}$, as shown in Equation (2). Let i=k,…,N and $f_{i},f_{i+2},\ldots f_{N}$ be the sub-branches of CFIU-k; then,

(2) $$\begin{array}{cc} \; & \{f_{i},f_{i+1},\ldots f_{N}\}=\\ \; & \delta \cdot \lambda \cdot Conv_{3}\cdot \Psi \left(CF_{k+1},DF_{k},DF_{k+1},\ldots ,DF_{N}\right)\\ \; & p_{k}=concate\left(CI,f_{i},f_{i+1},\ldots ,f_{N}\right), \end{array}$$

where symbols Ψ,λ,δ, and $Conv_{3}$ denote bi-linear interpolation, BN, ReLU, and (3×3) convolution, respectively. $CF_{k}$ and $DF_{k}$ denote the CFIUs and DFEUs at the kth stage, respectively.

To approach additional scale-specific features, the feature maps $p_{k}$ are first downsampled into $\bar{fi},\bar{f_{i+1}},\ldots ,\bar{f_{N}}$, distinct sub-resolutions using a soft attention mechanism [62], as shown in Figure 4 for CFIU-4. Each sub-stage of CFIU-k has a spatially separable convolution applied with large kernel sizes, i.e., (15×1) and (1×15) followed by a (1×1) and softmax operations. The softmax operation on the resulting feature maps empowers the attention mechanism using a residual connection to focus only on essential regions. These attention maps of each sub-branch are bilinearly upsampled to the size of the corresponding CFIU-k stage. However, as discussed in [61], the stack of refined attention maps often contains more numbers near to zero, making back-propagation difficult. To avoid this, we use a residual connection [63] to integrate each sub-branch feature map with their original feature maps $f_{i},f_{\left(i+1\right)},\ldots ,f_{\left(N\right)}$ and then reshuffle [60] the channels of each sub-branch to further increase their capabilities. Mathematically this process can be expressed as follows:

(3) $$\begin{array}{cc} \; & \{\bar{f_{i}},\bar{f_{i+1}},\bar{f_{N}}\}=\\ \; & \Psi \cdot \Phi \cdot Conv_{1}\left(\delta \cdot \lambda \cdot SSconv\left(\Pi \left(f_{i},f_{i+1},\ldots ,f_{N}\right)\right)\right)\\ \; & \;\;\;\;\;\;\;\;\;+\;\{f_{i},f_{i+1},\ldots ,f_{N}\}, \end{array}$$

where $\Pi ,\lambda ,\delta ,\Phi ,\Psi ,Conv_{3}$, and $Conv_{1}$ denote downsampling, BN, ReLU, softmax, bilinear interpolation (3×3), and (1×1) convolutional operations, respectively. SSconv denotes spatially separable convolutions.

All sub-branches of Equation (3) are summed and then subjected to a (3×3) convolution with BN and ReLU functions. The resulting feature maps are combined again with feature DFEU−k to update and enhance their overall capabilities, and a (1×1) convolution is then applied on the resulting feature maps to obtain the final refined feature maps, i.e., $W_{k}$ as shown in Equation (4).

(4) $$\begin{array}{cc} \; & W_{k}=Conv_{1}\left(\sum \left(\bar{f_{i}},\bar{f_{i+1}},\bar{f_{N}}\right)+DF_{k}\right), \end{array}$$

### 3.4. Contour-Aware Saliency Refinement Unit (CSRU)

**Figure 5:**
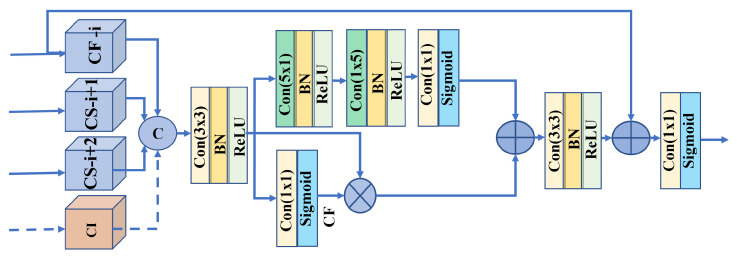
Contour-aware saliency refinement unit (CSRU), in which the supervised contour features (CFs) are integrated with salient features maps to guide the model towards more precise saliency maps with sharp edges. “CF−i” and “CS−i” indicate CFIU and CSRU modules, respectively.

The saliency maps can be directly generated after applying the CFIU by feeding delicate features into a basic set of convolution layers with a sigmoid activation function. However, owing to the high degree of variation between different stages, the accumulation process occasionally propagates additional noise in the case of challenging images, degrading the performance outcomes. We developed CSRU to address this problem by incorporating channel- and spatial-wise attention mechanisms. The proposed CSRU is a novel module that considers the low-level information to refine the edges and progressively extracts all high-level features by adopting light-weight convolution operations. For a better understanding, we visualized CSRU−3 in Figure 5. The CSRU retrieves the feature maps from their associated CFIUs via short connections for each decoder level. The proposed CSRU extracts CI from low-level resolutions. The various composite feature maps obtained from CFIUs and CI modules are combined to create highly discriminative feature maps. Subsequently, the integrated features are divided into two distinct narrow channel- and spatial-wise branches. The channel-wise unit extracts the most salient regions from a foreground object. It filters the integrated features for the most prominent areas as foreground objects using a (5×1), (1×5) spatial separable convolution and a (1×1) spatial separable convolution, followed by BN, ReLU, and a sigmoid operation with a residual connection.

Moreover, the second branch uses spatial-wise attention with a simple (1×1) convolution followed by a sigmoid function. It offers contour-based supervision to eliminate unnecessary background regions and maintain only significant contour information. After combining the two branching features using simple element-wise addition, (3×3) convolution, BN, and ReLU functions are applied. The resulting feature maps are combined with feature CF−i via a residual connection to empower the features associated with a certain stage at a given moment. The CSRU generates a saliency map by applying deep supervision at each stage and progressively upsamples the feature maps to merge with their subsequent adjacent resolution in the top-down pathway. The entire procedure is expressed in Equation (5):

(5) $$\begin{array}{cc} \; & R1=\gamma \cdot \delta \cdot Conv_{3}\left(\zeta \left(CF_{i},CS_{i+1},CS_{i+2},CI\right)\right),\\ \; & R2=\gamma \cdot \delta \cdot SSconv\left(\gamma \cdot \delta \cdot SSconv\left(R1\right)\right),\\ \; & R3=\sigma \cdot conv_{1}\left(R1\right)*R1,\\ \; & R4=\sigma \cdot Conv_{1}\left(\gamma \cdot \delta \cdot Conv_{3}\left(R2+R3\right)+CF_{i}\right), \end{array}$$

where ζ,δ,γ, and σ denote the channel-wise concatenation, BN, ReLU, and Sigmoid operations, respectively. $Conv_{3}$, $Conv_{1}$, and SSconv denote the (3×3), (1×1), and special separable convolutions, respectively. ∗ and + are element-wise multiplication and addition operations, respectively.

### 3.5. Hybrid Loss Function

To train our network and supervise feature learning in the two CSRU branches, we used the labels for the salient regions and boundaries. In saliency detection tasks, the loss of binary cross-entropy (BCE) is widely utilized as

(6) $$L_{bce}=-\sum_{x,y}G_{x,y}\cdot log\left(S_{x,y}\right)+\left(1-G_{x,y}\right)\cdot log\left(1-S_{x,y}\right),$$

where $G_{x,y}\epsilon \left(0,1\right)$ are the ground truth values at location (*x*, *y*), and S(x,y) ϵ[0,1] represent the saliency values at location (x,y) in their corresponding output prediction. However, the BCE loss estimates only the foreground loss for each pixel exclusively without considering the authenticity of the entire pixel set. To compensate for this, we additionally employ IOU loss [25,64] to calculate the similarity loss between two-pixel sets.

(7) $$L_{iou}=1-\frac{\sum_{x=1}^{H}\sum_{y=1}^{W}\left(S_{x,y}\cdot G_{x,y}\right)}{\sum_{x=1}^{H=1}\sum_{y=1}^{W}\left[\left(S_{x,y}+G_{x,y}\right)-\left(Sx,y\cdot G_{x,y}\right)\right]}.$$

Hence, we use the BCE loss function, i.e., $L_{Contour}$ for contour-based detection, and for SOD, we integrate the $L_{iou}$ and $L_{bce}$ loss functions:

(8) $$L_{sal}=\left(L_{i}^{bce}+L_{i}^{iou}\right).$$

The proposed total loss of our model is calculated by summing the losses of the two tasks as follows:

(9) $$L_{sal}^{T}=\sum_{i=1}^{5}\left(L_{sal}\right)+\sum_{i=1}^{6}\left(L_{Contour}\right).$$

## 4. Experiments

### 4.1. Datasets and Evaluation Metrics

We performed experiments to evaluate our AWANet model based on five publicly available datasets: ECSSD [30], HKU-IS [40], DUT-OMRON [32], PASCAL-S [65], and DUTS [66]. DUTS is the largest dataset with complicated scenes of 10,553 images for training and 5019 images for testing purposes. The ECSSD dataset contains 1000 natural-scene complex images. HKU-IS contains 4447 complex scene images having multiple disconnected images with a similar background or foreground. DUT-OMRON contains 5168 diverse images with complicated and cluttered backgrounds. PASCAL-S consists of 850 images with more challenging images chosen from PSCAL-S. Table 1 provides a short description for each dataset. We used five different evaluation techniques to compare our model’s state-of-the-art performance. These evaluation metrics include the precision-recall (PR) curve, maximum F-measure (maxF), S-measure (SM), and mean absolute error (MAE). To calculate the precision curve, we binarized the grayscale prediction maps using a fixed threshold. The generated binarized saliency maps and ground-truth masks were used to determine the precision and recall pairs; that is, Precision $=\frac{TP}{\left(TP+FP\right)}$ and Recall $=\frac{TP}{\left(TP+FN\right)}$, where TP, FN, and FP indicate true positives, false negatives, and false positives, respectively. A group of precision and recall scores were calculated to plot the PR curve when the threshold ranged from 0 to 255. A larger region under the PR curve indicates a better performance. The SM [67] calculates the region and object-aware structural resemblances denoted as Sr and So, respectively, between the predicted maps and ground-truth masks. The SM can be expressed as $\alpha \cdot S_{o}+\left(1-\alpha \right)\cdot S_{r}$, where the value of α was set to 0.5.

### 4.2. Implementation Details

We trained our model using the DUTS-TR [66] dataset by following the training protocols of [46,51,68]. All experiments were performed using a single Nvidia GTX TITAN X GPU. We allowed both vertical and horizontal flipping and image cropping to alleviate overfitting. We used the PyTorch framework to train and test the proposed model. We resized both the training and testing images to 320×320 px. For a reasonable evaluation, we chose VGG-16 [69] and ResNet-50 [63] as backbone networks. All weights of the convolution layers were arbitrarily initialized with a truncated normal (σ=0.01), and the corresponding biases were initialized to 0. The hyper-parameter weight decay and momentum were set to 0.0005 and 0.9, respectively, using the stochastic gradient descent (SGD) optimizer. The mini-batch was set to 18, with 40 epochs and without validation for training convergence. During inference, salient edge and saliency maps can be obtained. We summed the prediction maps as the final saliency map.

### 4.3. Comparison with State-of-the-Art Methods

We compared the proposed algorithm with 15 state-of-the-art saliency detection methods based on ResNet-50 and VGG-16 backbones on the five datasets. The comparison methods were NLDF [45], Amulet [70], SRM [51], PiCaNet [18], R3Net [23], BASNet [64], CPD [21], UCF [71], DSS [44], AFNet [22], EGNet [25], PAGENet [26], F3Net [72], MINet [73], CSB [74], PoolNet+ [75], and CAGNet [53]. To ensure a reasonable comparison, the proposed method was analyzed based on five different evaluation techniques.

**Figure 6:**
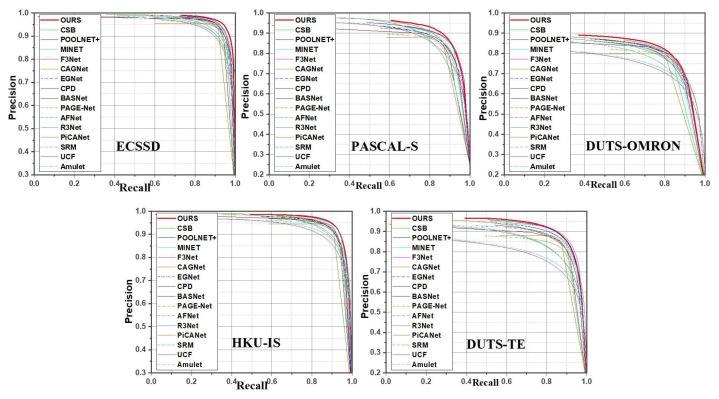
PR curve comparison on five different datasets.

**Figure 7:**
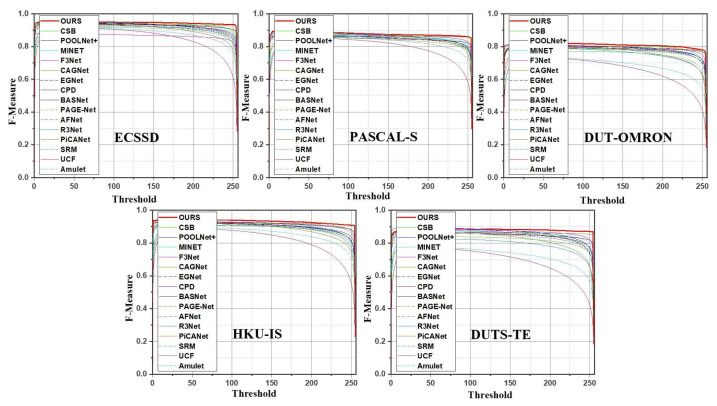
F-measure comparison on five different datasets.

**Quantitative Comparison**: To evaluate AWANet with the state-of-the-art methods, Table 2 lists the experiment results in terms of three metrics: maxF, SM, and MAE. As the results indicate, our approach exhibits a good performance and considerably outperforms the other methods, despite some approaches [23,44] using CRF [76] as a post-processing method. Our proposed method consistently achieves improved results, compared with the other models, across all five metrics on almost all datasets. Figure 6 and Figure 7 show the P-R and F-measure curves for the five testing datasets, respectively. Our method (solid red line) outperforms the other methods on the ECSSD, PASCAL-S, and DUTS-TE datasets and is notably superior on HKU-IS and DUT-OMRON. Another exciting aspect of the proposed model is that it requires fewer parameters (i.e., 26.7 M), which benefits applications with limited memory. We visualized our model’s comparison with state-of-the-art methods, as shown in Figure 6. Furthermore, the proposed model shows a speed above 31 FPS running a 384×384 image, which implies that our model can be used for real-time applications.

**Figure 8:**
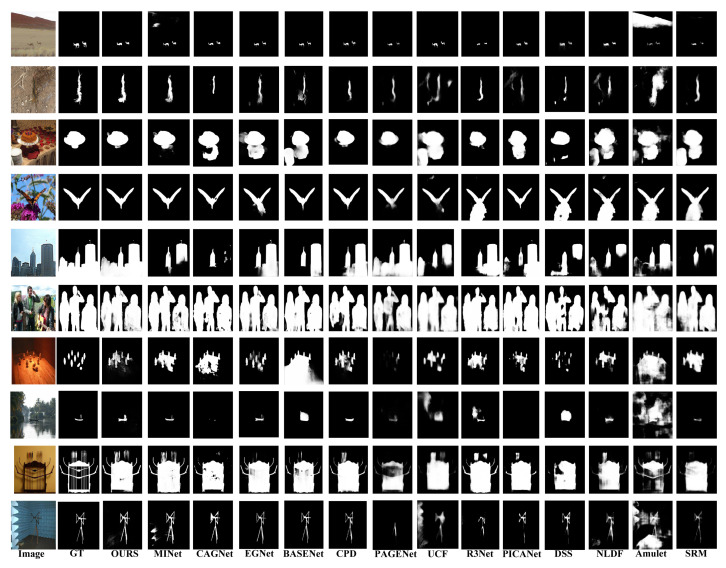
Visual comparisons with state-of-the-art methods in various circumstances: low contrast, complicated scene, large objects, small objects, and multiple objects.

**Qualitative Evaluation**: Figure 8 presents a visual comparison of our model’s representative examples with competitive methods. For a better visualization, we highlighted the critical complications of each image group. We observed that the proposed model performs well in various challenging scenarios. For example, in Figure 8, the first and second rows show the capability, in which the image has a low contrast between the object and its background. The third and fourth rows display images with cluttered environments. The 5th and 8th rows indicate the capability for the localization of small and large-scale objects.

Similarly, the sixth and seventh rows show multiple disconnected objects, and rows nine and ten show thread-like objects. Figure 8 verifies that AWANet generates more precise and complete saliency maps. Moreover, note that our model performs exceptionally well in capturing salient boundaries, owing to the additional CSRU.

### 4.4. Ablation Study

**Figure 9:**
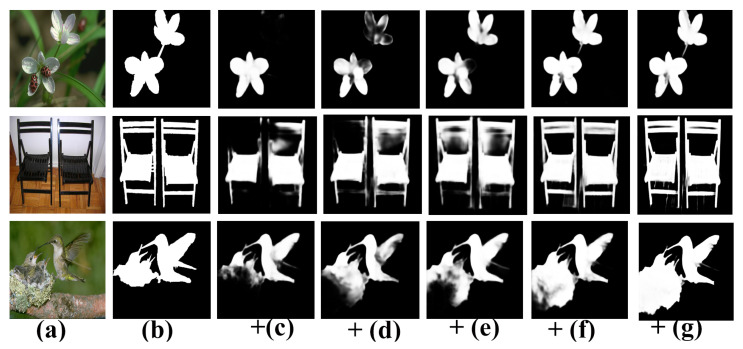
Examples of visual comparisons among the different modules of the proposed model: (**a**) input image, (**b**) ground truth, and (**c**–**g**) are saliency maps generated by the baseline (FPN), DFEUs, CFIUs, CSR, and CSR *.

**Figure 10:**
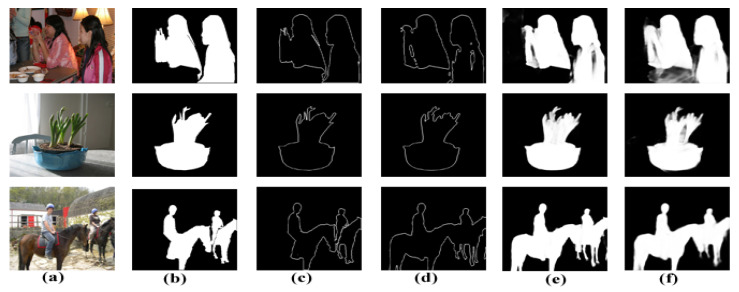
Saliency maps with and without contour-based supervision: (**a**) input Image, (**b**) ground-truth, (**c**) contour-based ground-truth, and (**d**–**f**) are the contour, saliency, and joint-supervision saliency maps generated by the CSRU, respectively.

This section analyzes the effectiveness of the different modules used in our method by conducting a detailed ablation study. We performed the experiments using DUT-OMRON [32] and DUTS-TE [66]. For the ablation analysis of the remaining modules, we considered the ResNet-50 FPN-like architecture [48] to be our baseline network. We performed an ablation assessment by progressively appending each of our modules to the baseline network. Table 3, Table 4 and Table 5 present the results in terms of the maxF, SM, and MAE evaluation metrics, respectively. Figure 9 and Figure 10 visualize the corresponding qualitative improvements.

**Effectiveness of the Dense Feature Extraction Unit (DFEU):** To validate the effectiveness of the proposed DFEU module, we appended this module to the last four levels of the encoder side. We ignored the first level in terms of appending subsequent modules because its large dimension rapidly increases the parameters and computational power without substantial impact. Table 3 summarizes the empirical results for the DFEUs, and Figure 9d visualizes predicted saliency maps. To further study the underlying details, we first replaced the convolution layers of the GCB block (shown in Figure 3) with trivial (3×3) convolution layers. Table 3 presents the relevant results across B+DFEUs-S. In the subsequent step, we replaced the convolution layers of the GCBs with asymmetric convolutions (spatially separable convolutions) using large kernels at various stages. We utilized the large kernels for the GCB, such as (3,5), (5,7), (7,9), and (9,11) for baseline stages 2 to 5, which can yield the best scores. The results listed in Table 3 for B+DFEUs-D indicate the enhanced performance of large kernels with dense receptive fields. We presented the results for the channel shufflings of Figure 3 in Table 3 in terms of the B+DFEUs and observed a slight performance gain. To further describe the originality and efficacy of the proposed DFEUs, we compared them with the most prevalent multi-scale modules: ASPP [24], and PPM [20]. As shown in Table 3, a significant drop in performance is observed if we replace the proposed DFEUs with ASPPs and PPMs. For DUT-OMRON, the DFEUs improve results to 0.796,0.824, and 0.059, in terms of the maxF, SM, and MAE compared with the baseline, respectively. Conversely, the ASPP and PPM improve the same metrics to 0.796, 0.818, 0.061 and 0.795, 0.822, 0.060, respectively. Hence, the DFEUs reduce the parameter size and perform better than PPM and ASPP modules.

**Effectiveness of the Cross Feature Integration Unit (CFIU):** The proposed CFIU module differs from other modules in the literature. First, it collects all matching high-level features via skip connections to learn a specific stage for different regions of a salient object. The aggregated features are then downsampled according to the number of input features received from the various stages via skip connections. Table 4 presents the ablation analysis results of the CFIUs to examine the efficiency of the internal components. The results labeled with “CFIUs w/o Att${}_{k=3}$” were obtained without sub-branch attentional or CI information with standard (3×3) convolutions. We observe a significant performance lead, i.e., 0.788, 0.816, and 0.060 against the baseline model in terms of the maxF, SM, and MAE. Similarly, the results presented in Table 4 for CFIUs w/ Att indicate the efficiency after embedding the spatially separable convolutions with channel-wise attention for each sub-branch of the proposed CFIUs. We performed multiple experiments with different kernel sizes (k = 7, 11, 15, and 21) and observed that the kernel size at k = 15 provides the optimum results. In addition embedded channel attention and large receptive fields further improve the performance efficiency to 0.799, 0.826, and 0.059 in terms of the maxF, SM, and MAE for DUT-OMRON against the baseline model. The embedded channel attentions in our CFIU module filter the challenging salient parts independently and then merge them, thereby guiding the model towards more accurate salient objects in scale-specific challenging environments. Furthermore, we present the impact of contour information integrated by the CFIUs in Table 4 with “CFIUs w/ CI” and observe a slight performance for each evaluation metric for the DUT-OMRON dataset. As the last row of Table 4, “B+DFEUs +CFIUs” indicates the overall performance gain of the CFIUs after the DFEUs and achieved an improved efficiency, i.e., 0.808, 0.836, and 0.056 in terms of the maxF, SM, and MAE, against the baseline model for the DUT-OMRON dataset. Compared with other feature integration modules in the literature, our CFIU is a lightweight module with fewer parameters that performs more robustly and expands only according to the given backbone levels in the top-down paradigm. For the visual results of the CFIU module, see Figure 9e.

**Effectiveness of the Contour-aware Saliency Refinement Unit (CSRU):** To verify the usefulness of the proposed CSRU, we applied it to each stage following the CFIUs. The CSRU is a novel lightweight module that collects all high-level features from their respective high levels via skip connections and solves the contextural feature dilution effect during top-down feature propagation. It then splits information into two branches after aggregating contextual and contour-based information. One branch generates contour maps utilizing the contour supervision in a spatial-attention manner. The other branch focuses on more foreground saliency information in a channel-attention manner. The CI directs the feature maps towards producing more exact and accurate saliency maps with sharper boundaries, as seen in Figure 9g and Figure 10e. Table 5 presents the quantified results of the proposed CSRU. CSR indicates the results obtained by integrating the proposed CSRU after DFEUs and CFIUs without contour-based supervision. In contrast, CSR * indicates results with CI. From the second row of Table 5, note that “CSRUs” with contour-based supervision (i.e., CSR *) provide the best results with a margin of 0.814, 0.840, and 0.054 against the baseline model, i.e., 0.775, 0.802, and 0.065 on DUT-OMRON.

**Effectiveness of Hybrid Loss:** We employed a simple hybrid loss function by introducing the IoU loss function with binary cross-entropy. We observe a slight performance gain in the results, i.e., 0.815, 0.842, 0.053 shown in the last row of Table 5 that indicate the importance of the proposed hybrid loss function. In particular, it performs best in MAE reduction.

**Memory and Speed Comparison:** The size of a deep learning model plays a significant role compared with other characteristics of deep learning SOD algorithms. Table 6 compares deep learning methods based on model size against the F-measure and MAE metrics on the DUTS-TE dataset. A model with fewer parameters, high F-measure, and low MAE can be considered optimal. Our model requires 26.7 M parameters and achieves an average speed time of more than 31 FPS on an i7 GPU and Titan X GPU, which is faster than most state-of-the-art models.

## 5. Conclusions

This study introduced AWANet, a novel end-to-end deep saliency model for SOD. The proposed model comprises three novel units: DFEUs, CFIUs, CSRUs. The DFEU extracts multi-scale features for each backbone level to accommodate scale variations in SOD tasks. The CFIU extracts multi-level contextual features via skip connections and leverages a stack of attention mechanisms to motivate the representative capability of the corresponding layer with multi-scale contextual features for precise and reliable SOD. The CSRU propagates salient contour details by learning accurate and precise boundary estimation to refine salient object segmentation. Experiments on five well-known benchmarks indicate the superiority of our proposed model against state-of-the-art methods.

## Figures and Tables

**Table 1:** Overview of the SOD-based datasets used to evaluate AWANet.

Datasets	Year	Images	Maximum Resolution	Object Properties	Difficulty	Subject Num-	Binary Classification
**ECSSD [30]**	2012	100	400 × 400	Single and large size objects	Simple background with disconnected objects	Manually annotated by five subjects	Yes
**PASCAL-S [65]**	2014	850	500 × 500	Multiple objects, moderate to large size	Simple background with complex structure objects	Manually annotated by 12 subjects	Yes
**DUT-OMRON [32]**	2013	5168	400 × 400	Multiple objects of a moderate size	Complex background with connected objects	Manually annotated by 25 subjects	Yes
**HKU-IS [40]**	2015	4447	400 × 400	Multiple objects from small to moderate in size	Complex objects with moderately challenging scenarios	Manually annotated by three subjects	Yes
**DUTS-TE [32]**	2017	5019	400 × 400	Multiple objects from small to large	Complex objects with complex and less contrastive background	Manually annotated by 50 subjects	Yes

**Table 2:** Maximum F-measure (maxF), S-measure (SM), and mean absolute error (MAE) of the proposed model and 15 state-of-the-art algorithms. ↑ and ↓ indicate higher maxF and SM values and lower MAE values indicate better outcomes. Top scores are in bold.

Method	Year	ECSSD			PASCAL-S			DUT-OMRON			HKU-IS			DUTS-TE		
Metric		maxF↑	SM↑	MAE↓	maxF↑	SM↑	MAE↓	maxF↑	SM↑	MAE↓	maxF↑	SM↑	MAE↓	maxF↑	SM↑	MAE↓
**VGG-16**																
**NLDF [45]**	2017	0.905	0.875	0.063	0.833	0.804	0.099	0.753	0.770	0.080	0.902	0.878	0.048	0.812	0.816	0.065
**DSS [44]**	2017	0.899	0.873	0.068	0.843	0.795	0.096	0.781	0.790	0.063	0.916	0.878	0.040	0.825	0.824	0.056
**Amulet [70]**	2017	0.914	0.912	0.059	0.850	0.831	0.099	0.742	0.784	0.098	0.895	0.914	0.051	0.777	0.803	0.085
**UCF [71]**	2018	0.903	0.884	0.069	0.825	0.807	0.115	0.730	0.760	0.120	0.888	0.874	0.061	0.631	0.782	0.112
**PiCANet [18]**	2018	0.931	0.914	0.046	0.871	0.851	0.077	0.794	0.826	0.068	0.922	0.905	0.042	0.851	0.861	0.054
**CPD [21]**	2019	0.936	0.910	0.040	0.873	0.843	0.074	0.794	0.818	0.057	0.924	0.904	0.033	0.864	0.867	0.043
**AFNet [22]**	2019	0.935	0.914	0.042	0.871	0.850	0.078	0.797	0.835	0.057	0.905	0.906	0.036	0.863	0.867	0.046
**PAGENet [26]**	2019	0.931	0.912	0.043	0.857	0.839	0.077	0.791	0.824	0.062	0.917	0.903	0.037	0.838	0.853	0.052
**EGNet [25]**	2019	0.942	0.912	0.040	0.867	0.846	0.077	0.808	0.835	0.056	0.923	0.906	0.035	0.876	0.877	0.044
**CAGNet [53]**	2020	0.930	0.897	0.040	0.857	0.825	0.073	0.782	0.806	0.057	0.926	0.903	**0.030**	0.851	0.850	0.043
**MINet [73]**	2020	0.943	**0.926**	0.036	0.883	0.854	**0.063**	0.810	0.833	0.053	0.928	0.910	0.031	**0.883**	0.874	**0.040**
**POOLNet+ [75]**	2021	0.941	0.917	0.040	0.874	0.857	0.070	0.806	0.836	0.056	0.874	0.852	0.036	0.886	0.876	0.042
**OURS**	2022	**0.945**	0.924	**0.035**	**0.886**	**0.865**	**0.063**	**0.814**	**0.842**	**0.052**	**0.935**	**0.912**	0.033	**0.887**	**0.879**	**0.040**
**ResNet-50/ResNet-101**																
**SRM [51]**	2017	0.917	0.895	0.054	0.850	0.866	0.064	0.769	0.798	0.069	0.906	0.887	0.046	0.826	0.836	0.059
**PiCANet [18]**	2018	0.935	0.917	0.046	0.870	0.855	0.064	0.803	0.832	0.065	0.919	0.905	0.044	0.860	0.869	0.051
**R3Net [23]**	2018	0.934	0.910	0.040	0.846	0.805	0.094	0.795	0.817	0.062	0.915	0.895	0.035	0.833	0.836	0.057
**BASNet [64]**	2019	0.942	0.916	0.037	0.863	0.837	0.077	0.805	0.836	0.056	0.930	0.908	0.033	0.859	0.866	0.048
**CPD [21]**	2019	0.939	0.918	0.037	0.872	0.847	0.072	0.797	0.825	0.056	0.925	0.906	0.034	0.865	0.869	0.043
**EGNet [25]**	2019	0.947	0.924	0.037	0.875	0.852	0.074	0.808	0.832	0.053	0.925	0.920	0.034	0.885	0.885	0.039
**CAGNet [53]**	2020	0.937	0.907	0.036	0.871	0.841	0.066	0.791	0.814	0.054	0.926	0.903	0.030	0.865	0.862	0.040
**F3Net [72]**	2020	0.945	0.924	**0.033**	0.882	0.860	0.067	0.813	0.838	0.053	0.936	0.917	0.028	0.888	0.886	0.036
**MINet [73]**	2020	0.947	**0.930**	**0.033**	0.878	0.855	0.063	0.809	0.833	0.055	0.935	0.918	0.028	0.886	0.883	0.037
**POOLNet+ [75]**	2021	0.948	0.926	0.035	0.887	0.865	0.065	0.805	0.839	0.052	0.922	0.913	0.035	0.888	0.887	0.037
**CSB [74]**	2022	0.944	0.921	**0.033**	0.885	0.860	**0.060**	0.811	0.834	**0.050**	**0.938**	0.918	**0.026**	**0.889**	0.879	**0.035**
**OURS**	2022	**0.949**	0.927	0.034	**0.889**	**0.872**	**0.062**	**0.815**	**0.842**	0.053	**0.938**	**0.921**	0.030	**0.889**	**0.895**	0.036

**Table 3:** Ablation analysis on the efficiency of the DFEU module. “B” refers to the baseline network, and “DFEUs-S” and “DFEUs-D” indicate DFEU modules of the same size with (3×3) kernels and with different large kernels. “B+DFEUs” indicates channel shuffling after “B+DFEUs-D”. The PPM and ASPP modules were compared by replacing the DFEU module. The best results are in bold.

Networks	DUT-OMRON			DUTS-TE		
	maxF	SM	MAE	maxF	SM	MAE
B	0.775	0.802	0.065	0.826	0.841	0.057
B+DFEUs-S	0.785	0.814	0.062	0.838	0.851	0.047
B+DFEUs-D	0.794	0.822	0.059	0.844	0.860	0.044
B+DFEUs	**0.796**	**0.824**	**0.059**	**0.846**	**0.862**	**0.043**
B+PPMs	0.795	0.822	0.060	0.843	0.858	**0.043**
B+ASPPs	**0.796**	0.818	0.061	0.845	**0.863**	0.044

**Table 4:** Ablation analysis concerning the efficiency of the CFIU module. “CFIU w/o Att”, “CFIU w/ Att”, and “CFIU w/ CI” show the CFIU module without an attention mechanism, with an attention mechanism by embedding the spatial separable convolutions with large kernels instead of the standard convolution, and with CI, respectively. We observe that the performance improves as the DFEU and CFEU modules are attached. The best results are in bold.

Networks	DUT-OMRON			DUTS-TE		
	maxF	SM	MAE	maxF	SM	MAE
B	0.775	0.802	0.065	0.826	0.841	0.057
B+CFIUs w/o Att${}_{k=3}$	0.788	0.816	0.060	0.843	0.860	0.045
B+CFIUs w/ Att${}_{k=7}$	0.796	0.821	0.060	0.851	0.869	0.043
B+CFIUs w/ Att${}_{k=11}$	0.797	0.824	0.059	0.853	0.872	0.043
B+CFIUs w/ Att${}_{k=15}$	0.799	0.826	0.059	0.855	0.873	0.042
B+CFIUs w/ Att${}_{k=21}$	0.798	0.825	0.059	0.853	0.874	0.043
B+CFIUs w/CI	0.801	0.827	0.058	0.856	0.875	0.041
B+DFEUs+CFIUs	**0.808**	**0.836**	**0.056**	**0.872**	**0.881**	**0.039**

**Table 5:** Ablation analysis on the effectiveness of the CSRU module. “CSR” refers to the CSRU without any CI supervision, and “CSR *” refers to the supervision of both saliency and CI. We observe that the performance improves as the different components of our model are attached. “DF”, “CF”, and “HL” are the abbreviations for the DFEU, CFEU, and hybrid loss, respectively.

Networks	DUT-OMRON			DUTS-TE		
	maxF	SM	MAE	maxF	SM	MAE
B+DF+CF+CSR	0.810	0.838	0.055	0.878	0.887	0.038
B+DF+CF+CSR *	0.814	0.840	0.054	0.886	0.892	0.037
B+DF+CF+CSR *+HL	**0.815**	**0.842**	**0.053**	**0.889**	**0.895**	**0.036**

**Table 6:** Ablation comparison with several known state-of-the-art methods in terms of the number of parameters in millions (#Par), average speed time (FPS), maxF, and MAE on the DUT-OMRON dataset.

Network	Network Size	Model Size (#Par)	FPS	DUTS-TE	
				maxF	MAE
Amulet [70]	256×256	31.6	16	0.8603	0.0512
Picanet [18]	226×226	37.02	7	0.8603	0.0512
BASNet [64]	400×300	87	25	0.8593	0.0483
EGENet [25]	400×300	108	14	0.8854	0.0390
POOLNet [77]	384×384	68.26	30	0.8832	0.0371
CSB [74]	384×384	27.9	32	0.8892	0.0346
F3Net [72]	384×384	26.5	31	0.8886	0.0359
CAGNet [53]	384×384	26.6	28	0.8659	0.0397
OURS	384×384	26.7	31	0.8894	0.0358

## Data Availability

Not applicable.
